# Imaging Techniques and Scanning Electron Microscopy as Tools for Characterizing a Si-Based Material Used in Air Monitoring Applications

**DOI:** 10.3390/ma9020109

**Published:** 2016-02-11

**Authors:** Beatriz Suárez-Peña, Luis Negral, Leonor Castrillón, Laura Megido, Elena Marañón, Yolanda Fernández-Nava

**Affiliations:** 1 Department of Materials Science and Metallurgical Engineering, Polytechnic School of Engineering, Gijón Campus, University of Oviedo, 33203 Gijón, Spain; 2 Department of Chemical and Environmental Engineering, University Institute of Industrial Technology of Asturias, Gijón Campus, University of Oviedo, 33203 Gijón, Spain; negralluis@uniovi.es (L.N.); cleonor@uniovi.es (L.C.); megidolaura@gmail.com (L.M.); emara@uniovi.es (E.M.); fernandezyolanda@uniovi.es (Y.F.-N.)

**Keywords:** imaging, scanning electron microscopy, microstructure characterization, Si-based materials, filtration behavior, air monitoring applications

## Abstract

This paper presents a study of the quartz fibrous filters used as a substrate for capturing the particulate matter (PM) present in the air. Although these substrates are widely used in environmental applications, their microstructure has been barely studied. The behavior of these devices during the filtration process was investigated in terms of their microstructure and the quartz fibers. Surface and cross sections were monitored. Scanning electronic microscopy with energy dispersive X-ray spectroscopy (SEM-EDX), imaging and stereology techniques were used as tools for this purpose. The results show that most of the quartz filter fibers have sizes that allow them to be classified as nanofibers. It was also observed that, while the mechanisms of the mechanical capture of particles via impaction, interception and diffusion operate simultaneously in the outer zones of the filter cross section, the mechanism of capture by impaction is virtually non-existent in the innermost zones. Particles between 0.1 and 0.5 μm are known to be the most difficult to have captured by means of fibrous substrates. The fibers in inner zones were highly efficient in capturing this type of particle.

## 1. Introduction

Fibrous filters are simple and economical devices capable of effectively capturing the submicrometric particles that are dragged by gas streams. The fibers used in filters of this kind can be made from a wide range of materials such as cellulose, glass, plastic, ceramics, and metals. This diversity allows their use in fields as varied as the manufacture of disposable respirators, the manufacture of industrial air cleaning equipment, the construction of air purification systems, the manufacture of air filtration systems for the automotive industry, and so on [1]. Glass and quartz fiber filters have been widely used as substrates for capturing particles in high volume samplers due, on the one hand, to their high efficiencies in capturing particles and, on the other, to the low resistances they display [2,3,4,5,6,7]. Numerous research papers have been published analyzing these filters. Some studies focused on the design of mathematical models intended to explain the mechanisms that operate during the capturing of particles. Happel [8], Kuwabara [9] and Spielman *et al.* [10] assumed in their calculations that the filters were constituted by a single size fiber. However, Brown *et al.* [11] considered a binary mix of fiber sizes, while Frising *et al.* [12] presented mathematical developments based on the size distribution of filter fibers. Several researchers have likewise analyzed the performance of these substrates during the capturing of particles. Sheng-Hsiu *et al.* [13] investigated the penetration of certain particle sizes through fibrous filters, while Podgórski *et al.* [14] demonstrated the behavior of filters made up of nanofibers. Furthermore, Marrero *et al.* [15] evaluated the homogeneity of the distribution of particles deposited on these filters, while Tuinman and Steenweg [16] analyzed the capacity of particles to penetrate these substrates depending on their morphology. However, although the behavior of filters during the filtration process depends on characteristics such as the size of their fibers, the size of the voids between the fibers, the number of contact points between fibers, *etc.*, only a few studies have investigated the microstructure of fibrous filters. Improved knowledge of the filtration process thus requires microstructural analysis of the filters used for collecting samples.

Stereology pursues the quantitative estimation of structural parameters based on two-dimensional planar cross sections of a material. The application of the principles of stereology enables the correlation of geometric aspects, such as points, lines, areas, and volumes, with the microstructural characteristics of filters, such as fibers, voids between fibers, and deposited particles [17]. Optical and electron microscopes are normally the tools of analysis used for quantification. Stereology is a method that uses a systematic random sampling method to provide systematic, unbiased information. A three-dimensional interpretation of the microscope images taken at random, obtained in planar cross sections of the substrates, provides a better understanding of the structure of fibrous filters [18]. Consequently, by using the principles of stereology, the main objective of this investigation has been the microstructural characterization of quartz fibrous filters and the analysis of their functionality during the capturing of airborne PM. The study was conducted not only on the surface but also throughout its cross section. Imaging and scanning electron microscope with energy dispersive X-Ray spectroscopy techniques were used as tools for this purpose. The results show that, while the mechanisms of the mechanical capture of particles via impaction, interception and diffusion operate simultaneously in the outer zones of the filter cross section, and the mechanism of capture by impaction is virtually non-existent in the innermost zones.

## 2. Materials and Methods

### 2.1. Filter Structure

**Figure 1:**
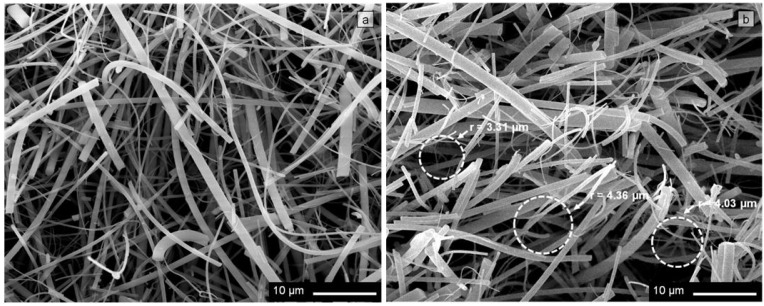
SEM images of a “clean” filter, in which it can be seen that it is made up of quartz fibers of different diameters. (**a**) Micrograph of the filter surface, in which the existence of points of contact between the quartz fibers can be appreciated; (**b**) micrograph of the transversal cross section of the filter in which voids can be observed surrounded by groups of quartz fibers, as well as several circumferences of variable radii between the fibers.

The efficiency of the filters employed in this study is based on the use of quartz fibers with different diameters that intertwine at random forming a structure of heterogeneous porosity (Tissue Quartz 2500QAT-UP, Pallflex, Port Washington, NY, USA). From the structural point of view, they can be considered three-dimensional filtration substrates. Figure 1 shows the micrographs corresponding to the longitudinal (Figure 1a) and transverse (Figure 1b) cross sections of a clean quartz filter. The surface area of the substrate is approximately 1.76 × 10^−2^ m^2^, its thickness is around 600 µm, and the mass/area ratio is 6.29 kg/m^2^.

### 2.2. Sample Collection

Sample collection was performed in Gijón, a city in northern Spain. A CAV-A/MSb sequential high volume sampler with mass flow control (MCV SA, Barcelona, Spain), capable of working with airflows of ~500 L/min and equipped with a selective inlet head for capturing particles with a 50% efficiency-cut-off at 10 µm aerodynamic diameter (PM10) was employed for this purpose. The sampling time was 24 h, after which time the quartz fibrous filter was removed for subsequent characterization by scanning electron microscopy.

### 2.3. SEM Analysis and Imaging

Samples extracted from a “clean” filter and from a filter after collecting particles belonging to the PM10 fraction were analyzed. The study of both quartz filters was conducted using a scanning electron microscope (SEM). Samples of ~1 cm^2^ were extracted. Substrates were directly “flat-mounted” on aluminum SEM stubs, using a two-sided adhesive film to adhere the filter to the stub, and were covered by a thin gold coating. Microscopic observation was carried out by means of a JEOL JSM 6100 SEM (JEOL Ltd., Tokyo, Japan), equipped with secondary electrons (SE) and backscattered electrons (BE) detectors able to provide detailed information on the morphologic characteristics and texture of the surface of the samples, as well as on the composition of particles observed in the image [19]. Chemical examinations were carried out with an energy EDX-system OXFORD INCA Energy 200 (OXFORD INSTRUMENTS Analytical Ltd., High Wycombe, UK). The microscope operated at an accelerating voltage of 20 kV, employing a beam spot size of 15. Given the heterogeneous nature of the filter, the images were obtained using a high contrast-to-brightness ratio to optimize the visualization of the particle edges [20]. The images were subsequently processed using the Kappa ImageBase image analysis software. This application, developed by Kappa optronics GmbH (Gleichen, Germany), is capable of processing images and creating image overlays, thus enabling the quantitative description of the major characteristics of the filters and the particles deposited on them, *i.e.*, volume fractions, sizes, morphologies, *etc.*

When the deposition of particles takes place on filters with homogenous structures and negligible thicknesses, e.g., polycarbonate filters, microscopic observations are limited to the surface of the substrates. Homogeneous deposition of the particles on the surface is assumed. A fraction of the surface area of the filter is microscopically analyzed in the direction perpendicular to the flow lines and the results are extrapolated to the entire filter [21,22]. However, when working with filters whose structure is both heterogeneous and three-dimensional, *i.e.*, fibrous filters, it will also be necessary to analyze the behavior of the substrate in the direction of the flow lines. In the present research study, the capturing of particles was evaluated both on the surface of the filter and through its transversal cross section. To this end, the transversal cross section of the filter was divided into four zones of analysis, henceforth referred to as deposition zones (DZs). These zones are located at different depths: 0, 100, 300 and 600 µm. The estimation of the amount of particles deposited in these DZs, *i.e.*, the volume of particles per unit volume, or volume fraction of particles, *V_Vp_*, allowed the determination of the degree of homogeneity in deposition through the cross section of the quartz fibrous filters. The volume of quartz fibers contained in the filter, *V_Vf_*, was also estimated. Determinations were carried out via the application of Quantitative Stereological Principles [23,24]. A manual point counting technique was used to obtain the volume fraction [25,26,27]. To perform this test, a grid composed of points was superposed over the microstructure. The grid consists of horizontal and vertical lines. The number of points that are inside the characteristic and those on characteristic boundaries (weighted as one-half a “hit”) is counted. This is repeated for N fields. The point fraction is then calculated and constitutes an estimate of the volume fraction. The sizes of the particles deposited at different depths and the sizes of the quartz fibrous filters were also estimated. The determinations were carried out manually, from the measurement of the Feret diameters (*d_F_*) of the particles and fibers [28]. The maximum distances between two parallel lines at a tangent to the contour of the characteristics were likewise measured. Although this is a directional estimate, it is sufficient, as the measured characteristics have random orientations.

The sizes of the existing voids between quartz fibers were likewise determined. To perform this test, superimposed circumferences were drawn between the quartz fibers observed in the SEM images (Figure 1b). The measurement of the radii of the circumferences, *r*, allowed us to estimate the size of the existing voids between the filter fibers [29,30].

The number of necessary observation sectors for the estimations was determined considering the number of characteristics observed in each area of visualization. More areas were analyzed in those cases in which a small number of characteristics were observed. At least 200 characteristics were analyzed in order to obtain a reasonable degree of accuracy [31,32].

### 2.4. Particle Deposition Mechanics

During the filtration process, the air will form flow lines dividing up the total airflow. Depending on its size, a particle dragged by the air towards the surface of the filter will follow the trajectory of the flow lines or not. When, due to its inertia, the particle is not able to adjust rapidly enough to the changes in airflow occurring on the surface of a fiber, it will impact against it and be retained [9,33]. This mechanism of particle uptake is called capture by inertial impaction. It is expressed by means of the Stokes number, *Stk*, which relates the stopping distance of the particle, *S*, to the diameter of the fiber, *d_f_*, both expressed in the unit of meter [34]:

(1) $$Stk=\frac{S}{d_{f}}$$

The Stokes number accordingly increases linearly with decreasing fiber diameter.

The stopping distance of a particle, *S*, may be calculated using the expression:

(2) $$S=\frac{\rho_{p}d_{p}^{2}{VC}_{c}}{18\mu }$$

where ρ*_p_* is the particle density, *d_p_* the particle diameter, *V* the filtering rate, and μ the gas viscosity. *C_c_* is the Cunningham correction factor, whose calculation is given by the following equation [35]:

(3) $$C_{c}=1+\frac{\lambda }{d_{p}}\left[2.34+1.05\text{exp}\left(\frac{-0.39d_{p}}{\lambda }\right)\right]$$

where *d_p_* is the particle diameter and λ is the mean free path of air molecules. Under standard conditions, λ is approximately 0.066 µm [34].

From the above Equations (2) and (3), the calculation of the Stokes number is:

(4) $$Stk=\frac{\rho_{p}d_{p}^{2}{VC}_{c}}{18\mu d_{f}}$$

When the particle approaches the fiber, Wang *et al.* [36] have shown that, if *Stk* >> 1, the particle will continue its path without deviating, finally colliding with the fiber. However, when *Stk* << 1, the particle will follow the flow lines, even when these are deflected around the fiber and therefore will not impact against it.

When a particle follows the path of the flow lines, the distance from its center of mass to the surface of the fiber may be equal to or less than its radius as it approaches a fiber. Under these circumstances, the particle will be captured by the fiber via a mechanism called interception [37]. This mechanism is defined by the ratio between the respective diameters of the particle and fiber, *d_p_* and *d_f_*:

(5) $$R=\frac{d_{p}}{d_{f}}$$

Using this relationship, Lee *et al.* [33] developed an approach for calculating the single fiber efficiency of the interception mechanism:

(6) $$\eta =\frac{1-V_{Vf}}{K}\cdot \frac{R^{2}}{1+R}$$

where *V_Vf_* is the volume fraction of the filter fibers and *K* is the Kuwabara hydrodynamic flow factor:

(7) $$K=-\frac{lnV_{Vf}}{2}-\frac{3}{4}+V_{Vf}-\frac{V_{Vf}^{2}}{4}$$

The smallest and lightest particles are mainly captured by the filter by means of diffusion. This mechanism has its origins in random motion, known as Brownian motion, to which smaller-sized particles are subject. This movement around the flow lines increases the likelihood of the particles coming into contact with the surface of the fibers. When the flow follows a pathway perpendicular to the alignment of the fibers, the efficiency of the filter in capturing particles by means of this mechanism, η, is given by the following equation [33]:

(8) η=2.6×(1−VVfK)13×Pe−23

where *V_Vf_* is the volume fraction of the fibers and *K* is the Kuwabara hydrodynamic flow factor (Equation (7)). *Pe* is the Peclet number, the parameter which governs convective Brownian diffusion and which relates the flow rate, *V*, and the coefficient of diffusion, *D*:

(9) $$Pe=\frac{d_{f}\times V}{D}$$

with the diameter of the fiber, *d_f_*. The Brownian diffusion coefficient of the particle, *D*, can be evaluated by means of the Stokes-Einstein expression. The calculation of *D* for a given temperature, *T*, is given by the equation:

(10) D=k×T×B

where *k* is the Boltzmann constant and *B* is a factor evaluating particle mobility. Assuming a spherical particle morphology:

(11) $$B=\frac{C_{c}}{3\pi µd_{p}}$$

where *C_c_* is the Cunningham slip correction factor (Equation (3)), µ is the viscosity of the gas and *d_p_* is the particle diameter.

## 3. Results and Discussion

Filters for capturing particulate matter behave differently depending on their chemical and morphologic characteristics [38]. Quartz fibrous filters are essentially collections of individual quartz fibers intertwined with one another giving rise to more or less integrated structures. In a system of this type, any external stimulus is transmitted either through the contact zones between the quartz fibers or between these and the medium filling the pores located between the fibers, *i.e.*, air [39]. Therefore, understanding the behavior of filters during the filtration process requires a prior study of this substrate on which the particles are collected. The analysis of the results relative to the process of deposition of aerosol particles on the quartz fibers of the filter was carried out following the classic theory of single fiber filtration [34]. Within this context, only the mechanisms of mechanical filtration are considered, *i.e.*, diffusion, interception and impaction. This section contains the interpretation of the results of the determinations and a discussion of the possible practical implications of the observations. The discussion focuses on two aspects:

- Characterization of the microstructure of quartz fibrous filters;
- Analysis of the behavior of quartz fibrous filters during the filtration process.

### 3.1. Characterization of the Microstructure of Quartz Fibrous Filters

In Figure 1, SEM images of the filters are shown. As may be observed, the filters under study are isotropic materials in which all of the quartz fibers are oriented randomly, without any preferential direction. The randomness of the orientation of the fibers means that the fiber density is independent of spatial coordinates. The behavior of a system of this type will depend, on the one hand, on the characteristics of the fibers and, on the other, on the relative amounts of fibers and voids between fibers. Both parameters determine the points of contact between fibers, as well as the free segments of fibers between two contact points [40].

**Figure 2:**
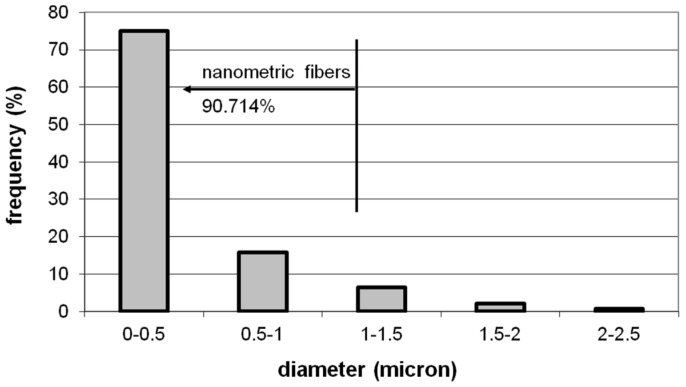
Size distribution of the quartz fibers found in the filters. According to these estimations, 90.714% of quartz fibers can be considered nanofibers.

Table 1 shows the mean values of the volume fraction of quartz fibers, $\bar{V_{Vf}}$, and the maximum, minimum and mean diameters of the quartz fibers, *d_f Max_*, *d_f Min_* and ${\bar{d}}_{f},$ respectively. Figure 2 shows the size distribution of the quartz fibers found in the filters. In general, nanoparticles are considered as those with a diameter of less than 0.01 µm (10 nm) [34]. However, fibers with diameters of less than 1 µm are considered nanofibers [14,41,42]. Accordingly, 90.714% of the quartz fibers in the filters under study may be classified as nanofibers (Figure 2), the fraction of fibers with diameters greater than 1 µm being limited in number.

**Table 1:** Quantitative determinations of the volume fractions of the quartz fibers, $\bar{V_{Vf}}$, at the 95% confidence level, and of the voids between quartz fibers, $\bar{V_{Vv}}$. Measurements of certain structural parameters of the filter: maximum diameter, *d_f Max_*, minimum diameter, *d_f Min_*, and mean diameter, ${\bar{d}}_{f}$, of the quartz fibers and maximum, *r_v Max_*, minimum radius, *r*_v Min_, and mean radius, ${\bar{r}}_{v}$, of the voids between quartz fibers.

Fibers					Voids			
$\bar{V_{Vf}}$ (%)	CL-_95%_ (%)	*d_f_* (µm)			$\bar{V_{Vv}}$ (%)	*r_v_* (µm)		
		*d_f Max_*	*d_f Min_*	${\bar{d}}_{f}$		*r_v Max_*	*r_v Min_*	$\bar{r_{v}}$
44.814	5.544	2.137	0.058	0.406	55.186	4.636	0.913	2.679

Given that transport processes take place through the interfaces of the substrate fibers [39], it is of interest to determine their specific surface areas:

(12) $$\frac{S}{V}=\frac{\pi d_{f}l_{f}+\pi \frac{d_{f}^{2}}{2}}{\pi \frac{d_{f}^{2}}{4}l_{f}}=2\left(\frac{2}{d_{f}}+\frac{1}{l_{f}}\right)$$

where *S* is the fiber surface area, *V* is the fiber volume, and *d_f_* and *l_f_* are the diameter and length of the fiber, respectively.

In the filters under study, quartz fiber diameters range between maximum values of 2.137 µm and minimum values of 0.058 µm, with a mean diameter of 0.406 µm (Table 1). According to Equation (12), the specific areas of the fibers decrease with increasing diameters. During the capturing of particles, there will be differences between the behavior of quartz microfibers and quartz nanofibers (Figure 1 and Figure 2). The latter, with a higher specific area, will show a significant increase in the likelihood of deposition of certain airborne particles on their surface [43,44].

Figure 1a shows a SEM image of the surface area of the filter in which the existence of points of contact between the quartz fibers can be appreciated. The characterization of the internal structure of the quartz filter requires determining the number of contact points that a fiber may establish with its neighbors, *n_l_*. The calculation of this parameter is given by the following equation [45]:

(13) $$n_{l}=\frac{8\;I\;V_{Vf}}{\pi d_{f}}$$

where I is a parameter dependent on the fiber orientation, *d_f_* is the fiber diameter, and *V_Vf_* is the volume fraction of fibers. According to Equation (13), for any given orientation, the number of contact points between filter fibers is independent of the length of the fiber, only depending on the diameter, *d_f_*, and the volume fraction of fibers of the substrate, *i.e.*, $\bar{V_{Vf}}$ = 44.814% (Table 1). Nanometric quartz fibers will contain more contact points than micrometric quartz fibers.

The quartz fibrous substrate is composed not only of fibers, but also of voids (Figure 1). Although the weight fraction of the air occupying these voids is small, their low density (~1 kg/m^3^) entails high volume fractions, *V_Vv_* = 55.186% (Table 1). The inherent limitations of filter processing techniques mean that the voids between the fibers are not uniform, not even those between fibers with the same orientation. The microscopic observations performed in this study have confirmed that the concentrations of quartz fibers and of voids between quartz fibers vary from one zone to another in the material. Furthermore, the voids in the filter are neither evenly distributed nor continuous, their areas varying from one zone to another of the substrate (Figure 1b). The application of image analysis techniques has allowed the determination of the dimensions of the voids in the filter, their ratios being found to range between *r_v Max_* = 4.636 µm and *r_v Min_* = 0.913 µm, the average value being $\bar{r_{v}}=2.679\;\mu \text{m}$ (Table 1).

### 3.2. Evaluation of Filtration Performance

**Figure 3:**
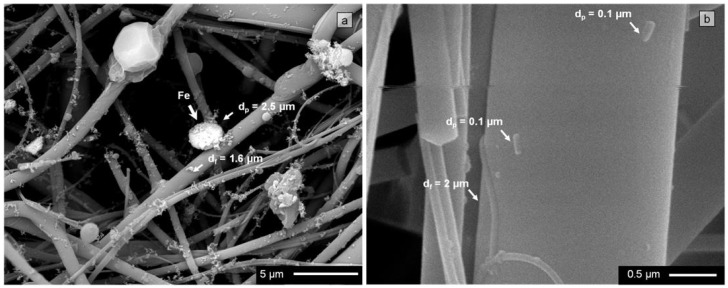
Quartz fibrous filter microstructure: (**a**) Backscattered electron image micrograph of filter surface. Quartz fibers of different diameters and captured particles of different sizes are observed. Those rich in Fe are brighter; (**b**) Filter cross section taken at a depth of 300 µm. Several particles can be observed deposited on a quartz fiber.

In the SEM image in Figure 3a, a Fe oxide microparticle (ρ*_p_* ~ 5.242 × 10^3^ kg/m^3^) of 2.5 µm in diameter can be observed, which is deposited on a 1.6 µm diameter quartz microfiber. During the filtration process, the microparticle was dragged by the air to the filter surface. The slip correction factor according to Equation (3) will be *C_c_* = 1.062. Considering an air viscosity μ = 1.983 × 10^−5^ Pa s and a flow rate during filtration of 8.333 × 10^−3^ m^3^/s, the filtration rate may have reached values close to 0.473 m/s. From Equation (4), the Stokes number will take a value of *Stk* = 28.817 (*Stk* >> 1). The Fe oxide microparticle therefore followed the path of the flow lines up to the vicinity of the quartz microfiber. However, it appears that inertia prevented it from adapting quickly enough to the variations in flow operating near the quartz fiber. It will have thus collided with the quartz fiber and been retained after impact (Figure 3a). Moreover, as the volume fraction of quartz nanofibers is the most abundant, *V_Vf_* = 90.714% (Figure 2), the collision may well have taken place against one of these fibers, e.g., *d_f_* ~ 0.1 µm. In this case, the particle would also have been trapped by means of this mechanism, as the Stokes number (Equation (4)) becomes *Stk* = 461.071 and thus *Stk* >> 1. However, if a Fe oxide particle of nanometric size (e.g., *d_p_* ~ 0.01 µm) were to collide in its path with the 1.6 µm diameter quartz microfiber, the Stokes number (Equation (4)) would be *Stk* = 9.975 × 10^−3^ (*Stk* << 1), whereas if the quartz fiber were a 0.1 µm nanofiber, *Stk* = 0.160 (*St* << 1). In both cases, therefore, the particle would follow the path of the flow lines without actually colliding against the quartz fibers. In short, quartz microfibers could be as effective as quartz nanofibers in capturing a Fe oxide microparticle via impaction. However, a nanoparticle of similar characteristics would not collide with the filter and would follow the flow lines, even when these are deflected around the given quartz fibers.

The effectiveness of the filter in capturing particles by means of the interception mechanism rapidly diminishes with decreasing particle size or increasing fiber diameter, as can be deduced from Equation (6). The most effective fibers in capturing particles by means of this mechanism correspond to the most abundant fraction in the filters, *i.e.*, quartz nanofibers (Figure 2). Lee *et al.* [46] determined that particles greater than ~0.5 µm in diameter are captured by fibrous filters by means of the mechanisms of interception and impaction, particle capture efficiencies via these mechanisms increasing with increasing particle size. Accordingly, both the above mechanisms may be excluded as likely mechanisms from capturing the particles indicated in Figure 3b. These are 0.1-μm diameter particles that have been retained by a 2-μm diameter quartz microfiber. The depth at which the particles are found (300 µm) suggests that they both passed through the filter surface without being captured by the quartz fibers in this zone, penetrating inside the cross section of the filter.

The coefficient of Brownian diffusion of the particle, *D*, is related to its diameter, *d_p_*, and to other parameters that are dependent on said diameter (10 and 11). In a first approximation, *D* can be assumed to be inversely proportional to the square of diameter of the particle, *d_p_*^2^ [11]. Thus, the smallest particles will present high diffusion coefficients, *D*, and small Peclet numbers, *Pe* (9). In brief, the filter will be efficient in capturing these particles by means of convective Brownian diffusion. Lee and Liu [33] have shown that particles of ~0.1 µm or smaller are captured by filters by means of the mechanism of diffusion, particle capture efficiencies by means of this mechanism increasing with decreasing particle size. This will hence be the most likely capture mechanism for the particles indicated in Figure 3b. It seems that these particles have advanced following the flow lines, although their small sizes indicate the possibility that the pathways of both describe Brownian motion around the flow lines. These random movements facilitated impact and, ultimately, the capture of both particles by the 2-μm diameter quartz fiber situated in the interior zone of the filter (DZ_300_).

McMurry [47] reported that there are intermediate size particles which are the most difficult to capture by means of fibrous filters. These are known as the most penetrating particles sizes (MPPS), which vary between 0.1 and 0.5 µm in diameter, depending on the characteristics of the filter and the flow passing through it. When the filter fibers work at relatively low filtration rates, they present minimal efficiency for capturing particles of around 0.3 µm in diameter [46]. Podgórski *et al.* [14] reported that a significant increase in filtration efficiency is achieved by nanometric fibers during the capturing of MPPS.

**Figure 4:**
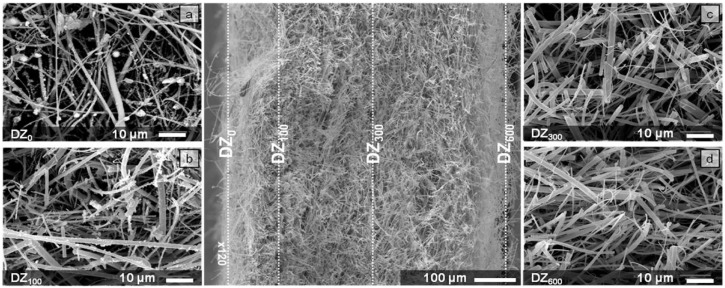
Backscattered electron image micrographs of the quartz fibrous filter cross section with highlighted zones discussed in more detail in the text. The boxes indicate the selected deposition zones (DZs) and their corresponding microstructure. (**a**) Deposition in DZ_0_, (**b**) Deposition in DZ_100_, (**c**) Deposition in DZ_300_, (**d**) Deposition in DZ_600_.

Figure 4 shows the particles captured in different zones of the cross section of the fibrous filter. The analyzed zones located at different deposition depths (DZs) are indicated in the micrograph located in center of the figure: 0, 100, 300 and 600 µm. It can be seen that only a few particles were captured in quartz fibers located in the more internal deposition zones of the filter, *i.e.*, DZ_300_ and DZ_600_ (Figure 4c,d), whereas the amount of particles captured by quartz fibers in the peripheral zone (DZ_0_) and close to this zone (DZ_100_) is quite significant (Figure 4a,b).

**Figure 5:**
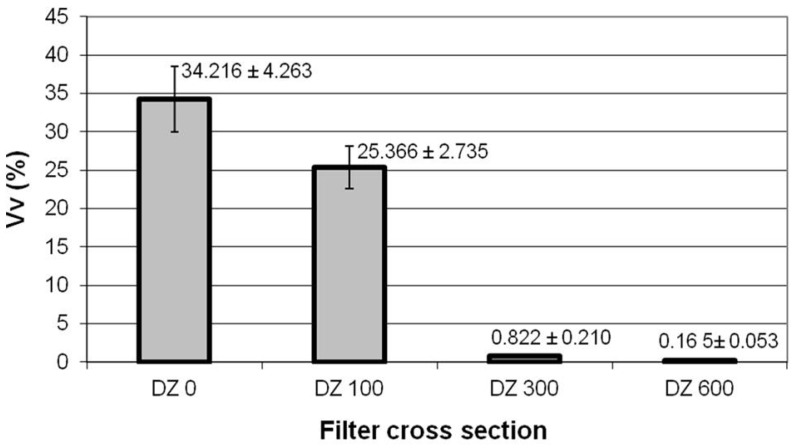
Quantitative determinations of the volume fraction, *Vv*, for PM10 particles conducted in selected zones of the cross section of the quartz filter. Error bars represent the 95% confidence limit of the determinations.

**Figure 6:**
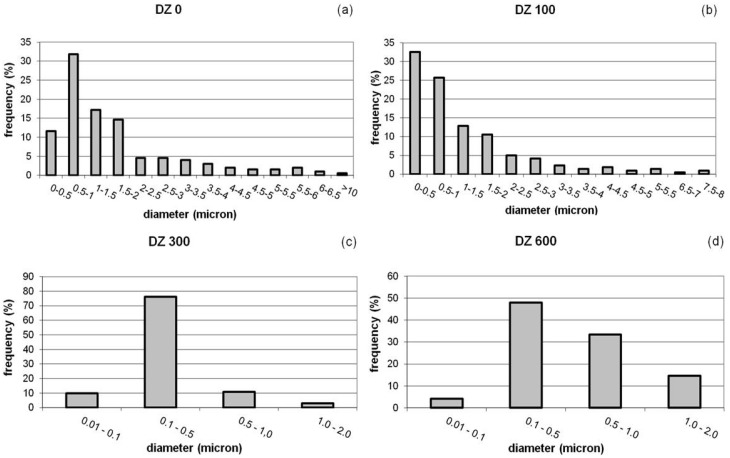
Particle size distribution for PM10 particles in selected zones of the cross section of the quartz filter: (**a**) on the free filter surface, DZ_0_; (**b**) at a depth of 100 µm, DZ_100_; (**c**) at a depth of 300 µm, DZ_300_; and (**d**) at a depth of 600 µm, DZ_600_.

Quantitative stereology and imaging techniques allowed us to quantify these observations. Figure 5, Figure 6 and Table 2 show the results thus obtained. The estimation of the volume fractions of the particles deposited in the different DZs is given in Figure 5. Figure 6 shows the results of the size distribution of the particles in the DZs, while the values of maximum particle diameter, *d_p Max_*, minimum particle diameter, *d_p Min_*, and mean particle diameter, $\bar{d_{p}}$, in each zone are given in Table 2.

**Table 2:** Values of the maximum, *d_p Max_*, minimum, *d_p Min_*, and mean particle diameters, $\bar{d_{p}}$, found in the different deposition zones in the quartz filter cross section.

DZ_0_			DZ_100_			DZ_300_			DZ_600_		
*d_p_* (µm)			*d_p_* (µm)			*d_p_* (µm)			*d_p_* (µm)		
*d_p Max_*	*d_p Min_*	$\bar{d_{p}}$	*d_p Max_*	*d_p Min_*	$\bar{d_{p}}$	*d_p Max_*	*d_p Min_*	$\bar{d_{p}}$	*d_p Max_*	*d_p Min_*	$\bar{d_{p}}$
10.272	0.199	1.682	7.760	0.075	1.280	2.000	0.050	0.311	1.849	0.058	0.533

In line with the above observations, Figure 5 shows that the zone with the largest volume fraction of particles captured by the fibrous filters is DZ_0_, namely, 34.216%. The volume of particles retained per volume unit of filter progressively decreases further inside the substrate. The quartz fibers in DZ_300_ and DZ_600_ captured hardly any particles, presenting volume fractions of 0.822% and 0.165%, respectively.

As regards to the size distribution of particles (Figure 6), the results show that, whereas the quartz fibers in DZ_0_ and DZ_100_ captured particles of a wide range of sizes, the quartz fibers in the innermost zones of the filter cross section, *i.e.* DZ_300_ and DZ_600_, retained particles with sizes ranging between 0.05 and 2 µm and from 0.06 to 1.85 µm, respectively (Table 2). Particles larger than 2 µm in diameter were not found in these interior zones, the particles of less than 1 μm in diameter being the most abundant in both DZs. Furthermore, the most abundant particle fraction in both zones is that corresponding to diameters of between 0.1 and 0.5 µm, *i.e.*, MPPS (Figure 6).

The results appear to indicate that the mechanisms of mechanical capture of particulate matter via impaction, interception and diffusion operate simultaneously in the outer zones of the cross section of the quartz filter, *i.e.*, DZ_0_ and DZ_100_, where high volume fractions and a wide range of captured particle sizes are observed.

However, the predominance of small particle sizes found in the innermost zones of the quartz filter cross section, *i.e.*, DZ_300_ and DZ_600_, seem to indicate that the mechanism of capture by impaction is virtually non-existent in these zones. Moreover, the quartz fibers in these interior zones are more efficient in capturing MPPS.

## 4. Conclusions

In this research study, fibrous filters have been characterized microstructurally and their behavior as a substrate for capturing the particulate matter present in ambient air (PM10) has been analyzed. The use of scanning electron microscopy and quantitative stereology and imaging techniques has allowed the characterization of the filters. The volume fraction of quartz fibers, their sizes and the voids between them have been determined. These results provide a better understanding of the behavior of filters during the process of filtering the airborne particles. The results show that:

1. 90.714% of the quartz fibers can be considered nanofibers, their diameters ranging between 2.137 and 0.058 µm.

2. The concentrations of quartz fibers and voids between fibers vary from one zone to another in the substrate. The voids in the filter are neither evenly distributed nor continuous. Furthermore, their sizes vary from one zone to another, from *r_v Max_* = 4.636 µm to *r_v Min_* = 0.913 µm.

3. Particle uptake through the cross section of quartz fibrous filter is not homogeneous, higher volume fractions of particles being found in the outermost areas of the cross section.

4. While the outer zones captured particles of a wide range of sizes, the innermost zones mainly captured particles below 1 μm in diameter. The quartz fibers in the inner zones were highly efficient in capturing particles considered difficult to capture, whose diameter ranged between 0.1 and 0.5 µm.

5. The results as a whole appear to indicate that the mechanisms of mechanical capture of particulate matter via impaction, interception and diffusion operate simultaneously in the outer zones of the cross section of the quartz filter. However, the mechanism of capture by impaction is virtually non-existent in the innermost zones of the filter cross section.
